# Relationship between *TNF-<alpha>* Gene Promoter Polymorphisms and Outcomes of Hepatitis B Virus Infections: A Meta-Analysis

**DOI:** 10.1371/journal.pone.0019606

**Published:** 2011-05-10

**Authors:** Qi Xia, LinFu Zhou, Dongcheng Liu, Zhi Chen, Feng Chen

**Affiliations:** 1 State Key Laboratory of Infectious Disease Diagnosis and Treatment, College of Medicine, First Affiliated Hospital, Zhejiang University, Zhejiang, China; 2 Department of Biochemistry, College of Medicine, Zhejiang University, Zhejiang, China; University of Rochester, United States of America

## Abstract

**Background:**

The clearance of hepatitis B virus (HBV) is a complex process which may be influenced by many factors including polymorphisms in the tumor necrosis factor *<alpha>* (*TNF-<alpha>*) gene promoter. However, previous reports regarding the relationship between polymorphisms in the *TNF-<alpha>* promoter and HBV clearance have been inconsistent. Therefore, we performed a meta-analysis on a large population to address this inconsistency.

**Methods:**

A meta-analysis was performed to examine the association between *TNF-<alpha>* promoter polymorphisms (-1031T/C, -863C/A, -857C/T, -308G/A and-238G/A) and chronic hepatitis B infection. Odds ratio (OR) and its 95 % confidence interval (CI) were used.

**Results:**

Twelve studies were chosen in our meta-analysis, involving 2,754 chronic HBV infection cases and 1,630 HBV clearance cases. The data showed that *TNF-<alpha>*-863 CC genotype was significantly associated with HBV clearance (-863 CC vs. AA: OR, 0.64; 95% CI, [0.42, 0.97]; p = 0.04) while patients carrying -308 GG genotype had a significantly increased risk of HBV persistence compared with those with GA or AA genotype (GG vs. GA+AA: OR, 1.35; 95% CI, [1.08, 1.70]; p = 0.01). For the other polymorphisms, no association with HBV infection outcome was found.

**Conclusions:**

The data showed that polymorphisms -863 A and -308 G in the *TNF-<alpha>* gene promoter region might be risk factors for HBV persistence. Furthermore, ethnicity might play an important role in HBV infection outcome, leading to conflicting results. More studies on individuals from various ethnic groups will be necessary to determine the role of *TNF-<alpha>* promoter polymorphisms in the outcome of HBV infection.

## Introduction

Approximately 5–10% of patients infected with hepatitis B virus (HBV) as adults are unable to clear the virus, ultimately developing chronic HBV infections[1]. The persistence of the virus is thought to be largely caused by a deficiency of the immune response to HBV[1]. The virus itself, environment factors, ethnic differences, and genetic susceptibility have also been reported to have some influence on the progression of this liver disease [2]. Recently, cytokine genetic polymorphisms have been found to be related factors that affect the progression of HBV infection [1].

Tumor necrosis factor *<alpha>* (*TNF-<alpha>*) is produced by macrophages, monocytes, neutrophils, T-cells and NK-cells after stimulation. In turn, *TNF-<alpha>* can stimulate cytokine secretion, increase the expression of adhesion molecules as well as activate neutrophils. Hence, it fulfills the role as a principal mediator of cellular immune response and inflammation, and may play an importance role in non-cytolytic and cytolytic clearance of HBV [3], [4], [5].

The *TNF-<alpha>* gene is located in the class III region of the major histocompatibility complex (MHC) on chromosome 6. The amount of cytokine production seems to be affected by the polymorphisms in the regulatory region. Therefore, there might be relationships between these single nucleotide polymorphisms (SNPs) and cytokine-mediated inflammation, which may affect the outcome of the disease.

There have been a number of studies on the association between chronic HBV infection (CHB), HBV clearance (HC), and *TNF-<alpha>* promoter polymorphisms -1031T/C, -863 C/A, -857C/T, -308G/A, and-238G/A. However, the results have been inconsistent. For instance, some studies indicated that patients carrying *TNF-<alpha>* genotypes (AA for -863, CC for -857, AA for -308, and AA for -283) have a higher risk of susceptibility to persistence of HBV [6], [7], [8], [9] while other studies did not[10], [11], [12].

A single study may fail to completely demonstrate this complicated genetic relationship because of a small sample size, which has low statistical power. Larger studies could overcome these disadvantages. Therefore, we performed a meta-analysis in an attempt to resolve this issue.

## Methods

### Search strategy

We searched the PubMed, EMBASE, ISI Web of Science, Google Scholar, Chinese National Knowledge Infrastructure Database and China Biological Medicine Database to collect all papers associated *TNF-<alpha>* polymorphism and HBV (last search update: 31^st^ July 2010). The following key words were used: “hepatitis B”, “HBV”, “Tumor necrosis factor *<alpha>*”, “*TNF-<alpha>*”, “polymorphism” and “SNP”. We also combined these key words to maximize the sample size in our analysis. *TNF-<alpha>* promoter polymorphisms -1031T/C, -863 C/A, -857C/T, -308G/A and-238G/A were investigated. The electronic searching was supplemented by checking reference lists from identified articles and reviews for additional original reports The language of the reviewed articles was limited to Chinese and English. Data were extracted by two authors independently and a consensus was achieved for all data. We excluded studies that were not full-length publications, and those that included no more than 10 participants. When study recruitment overlapped by more than 30% in two or more articles by the same author(s), the one with the largest population of participants or the most recent one was selected. We used Chi-square test to evaluate whether the observed frequencies of genotypes conformed to Hardy-Weinberg Equilibrium (HWE). CHB was defined as a condition in which serum HBsAg was positive for at least 6 months. HBV clearance (HC) was defined as a condition in which HBsAg was negative, but both HBV core antibody (anti-HBc) and HBV surface antibody (anti-HBs) were positive. None of the patients included in our study had any other type of liver disease such as hepatitis C or alcoholic liver disease.

### Data extraction

The following information was extracted by two authors independently, and a consensus was achieved: first author's name, year of publication, country, mean age of the study subjects, gender component, genotyping method, cases of CHB and HC with various genotypes, polymorphisms of *TNF-<alpha>* promoter.

### Statistical analysis

Odds ratios (ORs) with their confidence intervals (CIs) were calculated for each study. Heterogeneity was tested by chi-square-based Q test and I^2^ = 100%×(Q-df)/Q [13], [14]. The fixed effects model (Mantel–Haenszel method) [15] was used for calculating the pooled OR when the *P* value >0.05 for the Q test which indicated absence of heterogeneity among the studies. Otherwise, we used a random-effects model (DerSimonian-Laird method). Publication bias tests were performed by using the funnel plot, in which the standard error of log (OR) of each study was plotted against its log (OR). Funnel plot asymmetry was evaluated by Egger's linear regression test. A *P* value <0.10 was considered to indicate statistically significant publication bias.

## Results

### Extraction process and characteristics of the studies

**Figure 1:**
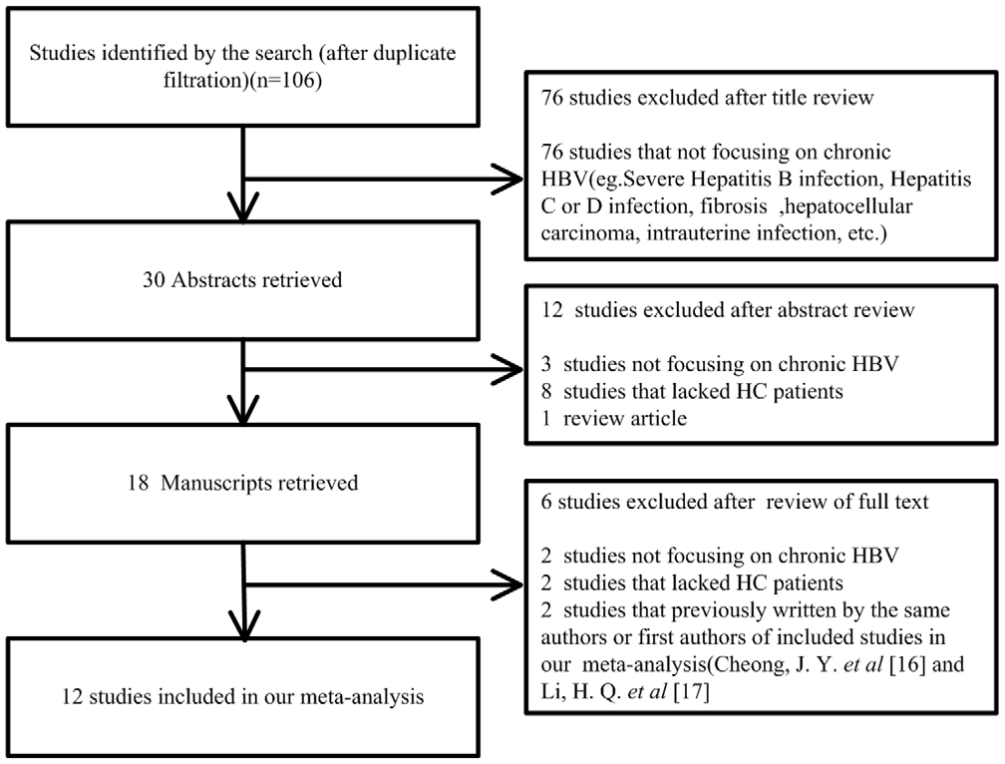
Flow diagram of identifying potential studies in our meta-analysis.

One hundred and six studies were identified after searching the databases. Seventy-six studies that not focused on chronic HBV(e.g. Severe Hepatitis B infection, Hepatitis C or D infection, liver fibrosis, hepatocellular carcinoma, intrauterine infection, etc.) were excluded after title review. Twelve studies were excluded after abstract review, three of them were not focusing on chronic HBV and eight lacked HC cases and one was a review article. After full text review, six studies were excluded, two of them not focused on chronic HBV and two were lacking HC cases and the other two were previously written by the same authors(Cheong, J.Y. *et al* [16] and Li, H. Q. *et al* [17]) of the rest studies, we selected the latest ones[10], [18]. The extraction process was showed in Fig 1.

Finally a total of 12 relevant studies were selected [6], [8], [9], [10], [11], [12], [18], [19], [20], [21], [22], [23] involving 2754 chronic HBV infection cases and 1630 HBV clearance cases. All studies included that the distribution of genotypes in the controls or recovered was consistent with Hardy-Weinberg equilibrium, except for two studies for -857C/T(Chen,D.Q *et al* [*6*])and -308G/A(Xing,P.X. *et al* [24]) .The included studies had been conducted on Chinese, Korean, Thai, Italian, Iranian, Brazilian and German participants. A summary of characteristics of these 12 included studies was showed in Table 1.

**Table 1:** Characteristics of Studies Included in the Meta-analysis.

Studies(Year/Countries)	Age (mean±SD)		Case of CHB(Males/%)	Case of HC(Males/%)	Methods	Studied Polymorphisms	Findings
	CHB	HC					
Hohler, T.[9](1998/ Germany)	NA	NA	71(NA)	32(NA)	PCR	−238,−308	The *TNF-<alpha>*−238 GG statistically significant differences between the CHB and recovered individuals
Kim, Y. J.[7](2003/Korea)	Male 49.9±10.4;Female 50.8±10.6	Male 54.7±10.9;Female 53.6±11.0	1109(74.4)	291(65.5)	Single base primer extension assay	−163,−238,−308, −857,−863, 1031	The *TNF-<alpha>*-308 AA or GA or -863 C/C were strongly associated with the resolution of HBV infection
Zhang,P.A [23](2004/China)	54.7±14.8	56.4±14.0	131(67.9)	165(66.1)	PCR-RFLP	−238,−308,−857 −863,	The *TNF-<alpha>*-308 A allele and -863 A allele may have a favorable effect in the clearance of HBV.
Li, H. Q.[17](2006/China)	33.37±12.67	34.68±11.34	433(81.8)	244(55.8)	PCR	−238, −857	The frequency of -238GG genotype in self-limited group was significantly higher than chronic HBV group .The frequency of *TNF-<alpha>*-857 CC in chronic HB group was significantly higher than self-limited group
Niro, G. A.[8](2005/Italy)	52±12	46.3±7.4	184(81.5)	96(79.2)	DNA sequencing	−238,−308, −863,−1031	The *TNF-<alpha>*-308GG was associated with an unfavorable prognosis in patients with chronic HBV infection.
Cheong, J. Y.[10](2006/South Korea)	37.74±8.90	47.96±s8.77	261(75.1)	204(72.5)	Single base primer extension assay	−238,−308	The *TNF-<alpha>* −308 and −238 were not different between the clearance and the persistence group
Somi, M. H.[12](2006/Iran)	NA	NA	100(NA)	91(NA)	PCR	−308	The *TNF-<alpha>*-308 A had no association with development of chronic HBV infection
Kummee, P.[20](2007/Thailand)	With HCC: 50.8±13.9	51.0±12.3	With HCC: 100(68.0)	100(48.0)	PCR-RFLP	−238,−308, −863	The *TNF-<alpha>*-863 A/A or A/C genotype were associated with increased the *TNF-<alpha>* levels in the liver in response to HBV infection and induced hepatocyte damage.
	Without HCC:57.5±14.2		Without HCC:50(60.0)				
Ribeiro, C. S.[11](2007/Brazil)	37±11.65	39.5±10.2	30(60.0)	41(48.8)	PCR	−308	The *TNF-<alpha>*-308 G/A had no association between chronic patients and self-limited infection
Xing,P,X[22](2007/China)	39.65±19.3	38.5±18.2	111(85.6)	100(66.0)	Gene Chips	−238,−308	The *TNF-<alpha>*-308 GG and G allele were higher in chronic HBV infection group than control group, but no association between *TNF-<alpha>*−238 and HBV infection was found.
Chen, D. Q.[6] (2010/China)	40.9±10.9	NA	252 (60.7)	109 (55.0)	PCR-RFLP	−238,−308,−857 ,−863	The *TNF-<alpha>*-308 G/G genotype was more frequent in patients than controls, while the frequencies of TNF -308 A/G genotype was higher in controls than in the patient group
Wan,P.Q[21](2010/China)	54.7±14.8	56.4±14.0	74(59.5)	64(62.5)	PCR-RFLP	−857, −863	In Guangxi population, the *TNF-<alpha>*-863 AA might go against the clearance of HBV.

Abbreviations: CHB, chronic HBV infection; HC, HBV clearance; HCC, hepatocellular carcinoma; PCR, polymerase chain reaction; PRLP, restriction fragment length polymorphism; NA, Not Applicable.

### Meta-analysis

Table 2 lists the results of the meta-analysis and heterogeneity test. Because the study by Kummee *et al* [20] contained a large number of individuals with HCC, subjects with or without HCC were included in our studies.

**Table 2:** Meta-analysis of effect of TNF-α promoter polymorphisms on the risk of HBV persistence.

	Genotype comparisons		NO. of CHB	NO. of HC	OR [95% CI,]	Z (*p* value)	Heterogeneity of study design		
							χ^2^	df (*P* –value)	I^2^
−1031	T allele *vs.* C allele	Overall	1617	495	0.86[0.68,1.08]	1.27(0.21)	0.01	1(0.90)	0%
	TT *vs.* CC	Overall	827	263	0.54[0.29,1.02]	1.89(0.06)	0.21	1(0.65)	0%
	TT *vs.* TC +CC	Overall	1222	379	0.82[0.65,1.05]	1.55(0.12)	0.06	1(0.81)	0%
	CC *vs.* TC +TT	Overall	1222	379	1.77[0.94,3.31]	1.78(0.08)	0.26	1(0.61)	0%
−863	C allele *vs.* A allele	Overall	2450	1387	0.91[0.78,1.07]	1.11(0.27)	8.40	5(0.14)	40%
		Asian	2208	1262	0.91 [0.77,1.08]	1.06 (0.29)	8.40	4(0.08)	52%
	***CC vs. AA***	***Overall***	***1312***	***743***	***0.64[0.42,0.97]***	***2.09 (0.04)***	***5.11***	***5(0.40)***	***2%***
		***Asian***	***1107***	***676***	***0.64[0.42,0.99]***	***2.01 (0.04)***	***5.10***	***4(0.28)***	***22%***
	CC *vs.* CA+AA	Overall	1881	1065	0.95[0.68,1.33]	0.29 (0.77)	17.06	5(0.004)	71%
		Asian	1697	969	0.97[0.65,1.44]	0.16(0.87)	17.06	4(0.002)	77%
	***AA vs. CA+CC***	***Overall***	***1866***	***1065***	***1.60 [1.06,2.41]***	***2.25(0.02)***	***6.21***	***5(0.29)***	***19%***
		***Asian***	***1682***	***969***	***1.60[1.05,2.45]***	***2.18(0.03)***	***6.21***	***4(0.18)***	***36%***
−857	C allele *vs.* T allele	Asian	1993	851	1.04[0.85,1.28]	0.38(0.70)	5.55	2(0.06)	64%
	CC *vs.* TT	Asian	1215	527	0.90[0.51,1.59]	0.35(0.73)	2.20	2(0.33)	9%
	CC *vs.* CT +TT	Asian	1604	689	1.13[0.69,1.85]	0.47(0.64)	9.47	2(0.009)	79%
	TT *vs*. CT+CC	Asian	1604	689	1.15[0.65,2.01]	0.47(0.64)	2.08	2(0.35)	4%
−308	***G allele vs. A allele***	***Overall***	***2175***	***1171***	***1.30[1.04,1.63]***	***2.30(0.02)***	***7.50***	***7(0.38)***	***7%***
		***Asian***	***1832***	***964***	***1.41[1.08,1.83]***	***2.56(0.01)***	***4.89***	***4(0.30)***	***18%***
		European	304	159	1.15[0.70,1.89]	0.56(0.57)	0.58	1(0.45)	0%
	GG *vs.* AA	Overall	2012	1178	0.62[0.23,1.67]	0.94(0.35)	0.98	6(0.98)	0%
		Asian	1785	1047	0.67[0.19,2.33]	0.64(0.52)	0.44	3(0.93)	0%
		European	206	97	0.35[0.04,2.95]	0.97(0.33)	0.02	1(0.89)	0%
	***GG vs. GA+AA***	***Overall***	***1967***	***1012***	***1.35[1.08,1.70]***	***2.59(0.01)***	***9.20***	***7(0.24)***	***24%***
		***Asian***	***1682***	***843***	***1.47[1.13,1.91]***	***2.85(0.004)***	***5.58***	***4(0.23)***	***28%***
		European	384	258	1.31[0.90,1.91]	1.41(0.16)	0.54	2(0.76)	0%
	AA *vs.* GA +GG	Overall	2271	1373	1.57[0.59,4.17]	0.90(0.37)	1.33	6(0.97)*	0%
		Asian	1836	1113	1.65[0.40,6.77]	0.69(0.49)	0.24	2(0.89)	0%
		European	384	258	1.49[0.47,4.71]	0.68(0.50)	0.26	2(0.88)	0%
−238	G allele *vs.* A allele	Overall	2715	1588	0.92[0.72,1.18]	0.65(0.52)	12.25	8(0.14)	35%
		Asian	2421	1451	1.01 [0.77,1.33]	0.10(0.92)	8.36	6(0.21)	28%
		European	294	137	0.50 [0.77,1.33]	1.87(0.06)	1.22	1(0.27)	18%
	GG *vs.* AA	Overall	2281	1384	0.88[0.32,2.45]	0.52(0.70)	0.52	4(0.97) *	0%
		Asian	2065	1265	0.83 [0.25,2.70]	0.23(0.75)	0.23	2(0.89)	0%
		European	216	119	1.07 [0.14,8.28]	0.24(0.95)	0.24	1(0.62)	0%
	GG *vs.* GA+AA	Overall	2498	1486	0.90[0.70,1.16]	0.42(0.42)	14.68	8(0.07)	46%
		Asian	2243	1358	1.01[0.77,1. 32]	0.07(0.95)	9.52	6(0.15)	37%
		***European***	***255***	***128***	***0.45[0.22,0.93]***	***2.15(0.03)***	***1.77***	***1(0.18)***	***44%***
	AA *vs*. GA +GG	Overall	2498	1486	1.08[0.39,2.98]	0.14(0.89)	0.66	4(0.96)	0%
		Asian	2243	1358	1.18[0.36,3.86]	0.27(0.79)	0.20	2(0.90)	0%
		European	255	128	0.81[0.11,6.16]	0.20(0.84)	0.34	1(0.56)	0

*-308 A/A and -238 A/A were rare genotypes. Therefore, some studies had to be excluded because they contained no individuals carrying these genotypes. In the study on -308 AA *vs*. GA +GG, the report by Kummee *et al* [20] was excluded; in -238 GG *vs*. AA and -238 AA *vs*. GA +GG, Cheong *et al* [10] and Li *et al* [17] were excluded.

Abbreviations: CHB, chronic HBV infection; HC, HBV clearance; OR, odds ratio; CI, confidence interval; df, degree of freedom.

**Figure 2:**
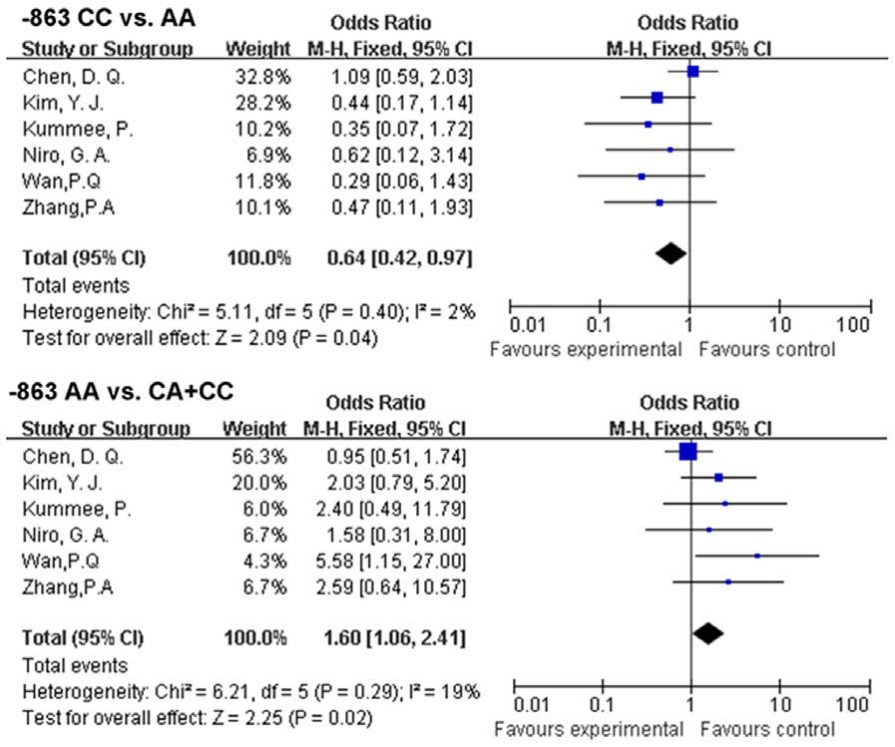
OR of HBV infection associated with *TNF-<alpha>*-863 C/A for the CC genotype compared with the AA genotypes and AA compared to the CA+AA genotype.

For *TNF-<alpha>*-1031T/C and, -857C/T, we found no association between the polymorphisms and HBV clearance. For *TNF-<alpha>*-863, overall, we found that compared to -863 AA,*TNF-<alpha>*-863 CC genotype was associated with chronic HBV clearance (-863 CC *vs.* AA:OR, 0.64; 95% CI, [0.42, 0.97]; p = 0.04) (Fig 2). When we excluded the subjects with HCC, the signification seemed to be weaken(OR, 0.66; 95% CI, [0.43, 1.00]; p = 0.05),implying that -863 CC might play a relatively different role in HCC patients. In a subgroup analysis of *TNF-<alpha>*-863 CC *vs.* AA by ethnicity, the pooled OR was significant in Asians (OR, 0.64; 95% CI, [0.11, 0.99]; p = 0.04). In addition, the result of AA *vs.* CA+CC model showed that -863 AA maybe a risk factor for HBV persistence (overall: OR, 1.60; 95% CI, [1.06, 2.41]; p = 0.02;Asian: OR, 1.60; 95% CI, [1.05, 2.45]; p = 0.02) which implied the counteractive function between -863 CC and -863 AA in virus clearance. A European subgroup analysis was not conducted because only one study [8] was available.

**Figure 3:**
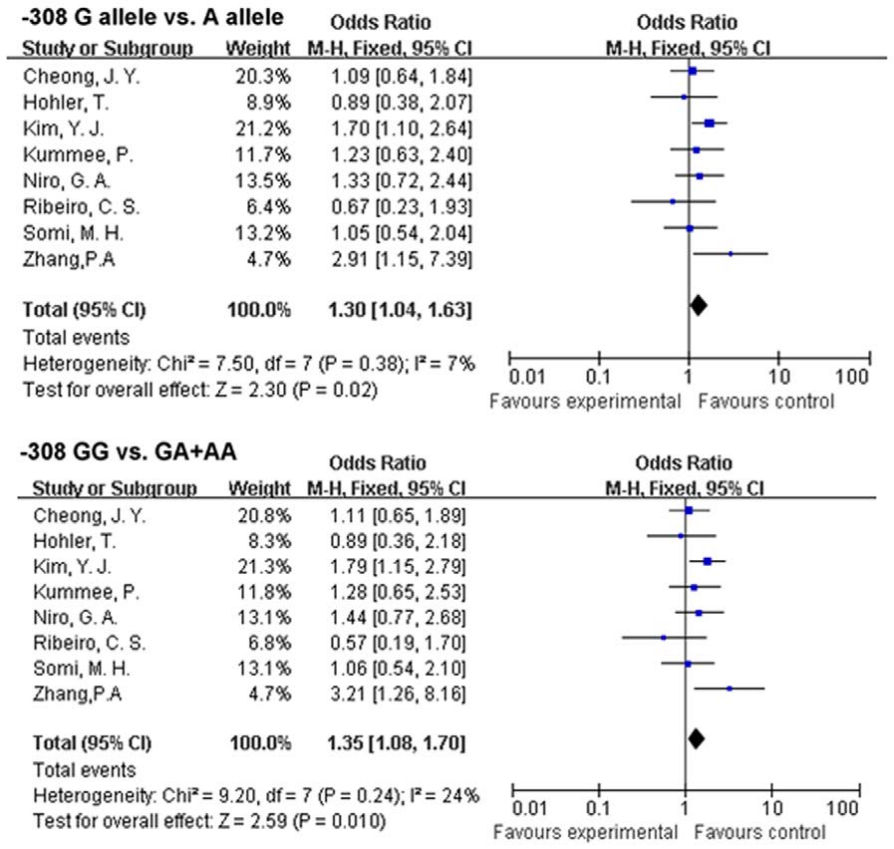
OR of HBV infection associated with *TNF-<alpha>*-308 G/A for the G allele compared with the A allele and the GG genotype compared with the GA+AA genotypes.

When we performed analysis on *TNF-<alpha>*-308 G/A we found two models had statistic significance(G allele *vs.* A allele: OR, 1.30; 95% CI, [0.104, 1.63]; p = 0.02 . GG *vs.* GA+AA: OR, 1.35; 95% CI, [1.08, 1.70]; p = 0.01) (Fig. 3) indicating that -308 G might be an unfavorable factor for the elimination of HBV. In subgroup analysis, the result of Asian group was similar (G allele *vs.* A allele:OR, 1.41; 95% CI, [1.08, 1.83]; p = 0.01; GG *vs.* GA+AA: OR, 1.47; 95% CI, [1.13, 1.91]; p = 0.004) while the European group lacked such an association. We also performed an analysis for East Asian individuals by removing a study conducting on Iraqi(Somi, M. H [12]) from Asian group , the result still stable(G allele *vs.* A allele:OR, 1.49; 95% CI, [1.12, 1.98]; p = 0.006; GG *vs.* GA+AA: OR, 1.56; 95% CI, [1.17, 2.08]; p = 0.002). When we excluded the subjects with HCC, the results remained similar(data not show).

Interestingly, significantly increased risks were observed in the European population (-238 GG *vs.* GA+AA: OR, 0.45; 95% CI, [0.22, 0.93]; *p* = 0.03), but not in the Asian population or overall analysis.

### Tests of heterogeneity

We found heterogeneities in three studies: (-863 CC *vs.* CA+AA, overall: χ^2^ = 17.06, *p* = 00.004, I^2^ = 71%; Asian: χ^2^ = 17.06, *p* = 0.002, I^2^ = 77%; -857 CC *vs.* CT +TT , χ^2^ = 9.47, *p* = 0.009, I^2^ = 79%) (Table 2). A random-effects model was employed in these studies.

### Publication bias

**Figure 4:**
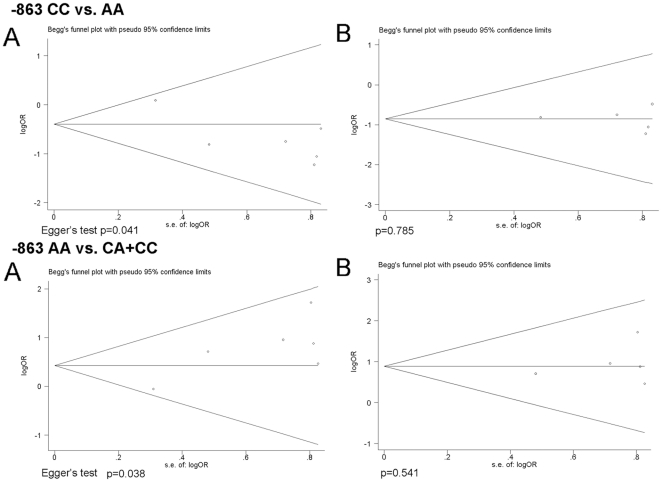
Funnel plot analysis to examine publication bias. Some asymmetry in -863 CC vs. AA (A) and -863 AA vs. CA+CC (A). After one study removed, the publication bias was eliminated. See -863 CC vs. AA (B) and -863 AA vs. CA+CC (B).

Begg's funnel plot and Egger's test were performed to access the publication bias of the studies. No evidence of publication bias showed in -308 G allele *vs.* A allele or -308 GG *vs.* AA model (funnel plot data not show, Egger's test p = 0.470 and 0.556,respectively). However, funnel plot showed some asymmetry in -863 CC *vs.* AA and -863 AA *vs.* CA+CC models(Fig 4) and Egger's proved the existence of publication in these two models(p = 0.041 and 0.038, respectively). We excluded one study from the meta-analysis to see whether the publication bias still presented. The results showed that after the exclusion of study Chen, D. Q. *et al* [6], the publication bias was eliminated(funnel plot showed in Fig 4, Egger's test -863 CC *vs.* AA:p = 0.785; -863 AA *vs.* CA+CC:p = 0.541),and the conclusion still stable(-863 CC *vs.* AA: OR, 0.42; 95% CI, [0.23, 0.77]; p = 0.005; -863 AA *vs.* CA+CC: OR, 2.44; 95% CI, [1.34, 4.45]; p = 0.003).

## Discussion

The mechanism of effective clearance of HBV from the human body is likely related to both environmental and host genetic factors. Several studies have reported that *TNF-<alpha>* plays an important role in HBV clearance. In an *in vitro* study, *TNF-<alpha>* was reported to be able to accelerate HBV mRNA destruction, and inhibit the replication of HBV[25]. An *in vivo* study also found that TNF limited chronic infection by destabilizing HBV nucleocapsids and reducing the cccDNA[26]. Furthermore, a clinical study showed that elevation of *TNF-<alpha>* levels in *IFN-<alpha>*treated patients led to HBV elimination[27].

In the current study, we performed a meta-analysis to examine the association between the SNPs in the promoter region of *TNF-<alpha>* and the outcome of HBV infection. According to our findings, the presence of G at the position -308 of *TNF-<alpha>* promoter gene polymorphisms increased the risk of HBV persistent infection significantly while -308 A may have a positive effect in virus clearance. Similar conclusion was drew by Zheng, M. H. *et al* [28] who also performed a meta-analysis focus on healthy individual(including spontaneously recovered case)and Chronic hepatitis B patient, they found that -308 A allele was a protective factor for CHB infection, especially in Mongoloid populations. Our analysis was restricted in CHB and HC case, thus our case size was smaller, but higher specificity would provide us more reliable evidence to our conclusion. Taken together, -308 A may play a crucial role in anti-virus mechanism in human body; it can not only protect healthy people from HBV infection but also enhance the scavengingof virus while being infected. This virus clearing function of *TNF-<alpha>* –308A allele was associated with the enhancement of *TNF-<alpha>* transcriptional activation as well as production [29], [30].

Our study showed that -863 CC and -863 AA might be another important factors in HBV clearance. Previous study reported that the -863 C/A performed their protective or deteriorative function for CHB infection through a difference way that -863 A allele can lower *TNF-<alpha>* promoter activity and plasma levels by weakening the affinity between specific protein and the segment of *TNF-<alpha>* promoter spanning from position −876 to position −845 [31].

Although the association between *TNF-<alpha>* and HCC patient has not been clearly understood, our studies gave some indirect evidence on a different response to the *TNF-<alpha>* in CHB and HCC individuals, for the discard of HCC cases from the analysis could affect the results, especially in -863 CC *vs.* AA model, thus, caution should be paid in this result and more investigations are demanded to interpret the relationship among *TNF-<alpha>* and HCC and CHB.

In addition, we found that genotype GG at position -238 of TNF-α promoter was associated with decreased risk of chronic HBV persistence in European populations which supports the study of Hohler *et al* [9]. However, the results differed from the study of Lu *et al* [32]. The current results suggest that ethnicity may have played an important role in HBV infection outcome, leading to results inconsistent with that of others. Further research is needed to demonstrate the underlying cause for this inconsistency.

In our study, other polymorphisms in the *TNF-<alpha>* promoter (-1031T/C,-857C/T) did not show any association with HBV outcome, which is different from some previous reports [7], [9], [17]. Possible explanations for this difference are a lack of data, and ethnicity diversity.

Our study provided a more believable result due to a larger size sample, and provides explanations for the inconsistencies observed in previous studies. Meta-analysis is a powerful statistical tool that provides a consensus by combining the data from diverse studies that reveal inconsistent results on the same problem. Some results of our study did not show any statistical significance although we combined relatively large numbers of studies. However, subgroup analysis by ethnicity showed a statistical significant result. Hence, a meaningful outcome can be produce only when it is correctly used. On the other hand, the more studies included, the more accurate the results would be.

In conclusion, this study provides evidence of a positive association between HBV clearance and *TNF-<alpha>* promoter -863 CC. Conversely, -308 GG/GG+GA genotypes increased the risk of chronic infection. These genotypes might affect the outcome of HBV infection through regulation of *TNF-<alpha>* transcriptional activation and production. Ethnic diversity may complicate the outcome of infection. More studies of individuals of diverse ethnicities will be necessary to determine the effects of *TNF-<alpha>* promoter polymorphisms on the outcome of HBV infection.
